# The Roles of Sclerostin in Immune System and the Applications of Aptamers in Immune-Related Research

**DOI:** 10.3389/fimmu.2021.602330

**Published:** 2021-02-25

**Authors:** Meiheng Sun, Zihao Chen, Xiaoqiu Wu, Yuanyuan Yu, Luyao Wang, Aiping Lu, Ge Zhang, Fangfei Li

**Affiliations:** ^1^Law Sau Fai Institute for Advancing Translational Medicine in Bone and Joint Diseases, School of Chinese Medicine, Hong Kong Baptist University, Hong Kong, China; ^2^Institute of Integrated Bioinfomedicine and Translational Science, School of Chinese Medicine, Hong Kong Baptist University, Hong Kong, China; ^3^Institute of Precision Medicine and Innovative Drug Discovery, HKBU Institute for Research and Continuing Education, Shenzhen, China; ^4^School of Chinese Medicine, Faculty of Medicine, The Chinese University of Hong Kong, Hong Kong, China; ^5^Institute of Basic Research in Clinical Medicine, China Academy of Chinese Medical Sciences, Beijing, China; ^6^Institute of Arthritis Research, Shanghai Academy of Chinese Medical Sciences, Shanghai, China

**Keywords:** sclerostin, Wnt signaling pathway, aptamers, immune system, B cell malignancies

## Abstract

Wnt signaling is one of the fundamental pathways that play a major role in almost every aspect of biological systems. In addition to the well-known influence of Wnt signaling on bone formation, its essential role in the immune system also attracted increasing attention. Sclerostin, a confirmed Wnt antagonist, is also proven to modulate the development and differentiation of normal immune cells, particularly B cells. Aptamers, single-stranded (ss) oligonucleotides, are capable of specifically binding to a variety of target molecules by virtue of their unique three-dimensional structures. With in-depth study of those functional nucleic acids, they have been gradually applied to diagnostic and therapeutic area in immune diseases due to their various advantages over antibodies. In this review, we focus on several issues including the roles of Wnt signaling and Wnt antagonist sclerostin in the immune system. For the sake of understanding, current examples of aptamers applications for the immune diseases are also discussed. At the end of this review, we propose our ideas for the future research directions.

## Introduction

Wnt signaling is one of the fundamental pathways that play a major role in a range of biological systems, such as stem cell development, tissue homeostasis, and immune cell modulation, the dysregulation of which is responsible for various disorders (1–3). Therefore, as a strong Wnt antagonist, the roles of sclerostin in the immune system have gained increasing research attention. Mechanistically, sclerostin executes its tasks in Wnt signaling pathway based primarily on competitively binding to Wnt co-receptors low-density lipoprotein receptor-related proteins 5 and 6 (LRP5/6) (4). Wnt-LRP5/6 dimers then form a trimer with seven-pass transmembrane Frizzled (Fz or Fzd) proteins to maintain the stability of *β*-catenin, a critical regulatory factor in the transcriptional function of Wnt signaling (5). Hence, a significant feature of sclerostin is its ability to mediate the developmental gene expression programs.

Regarding the roles of Wnt signaling pathway on B cells, divergent results were reported between mice and human. Wnt signaling cascade plays a central role in B cell development in murine fetal liver and bone marrow, while Wnt pathway acts as a negative regulator of proliferation potential of B cells in human bone marrow, which needs further investigation (6–8). However, when it comes to the roles of sclerostin on B cells, accumulating direct or indirect evidence suggests that sclerostin plays an indispensable role in normal B lymphocyte development. Absence of sclerostin resulted in enhanced B cell apoptosis and reduced CXCL12, a critical B cell growth-stimulating factor (9). Interestingly, loss of sclerostin in different osteolineage cells demonstrated differentially altered B lymphocyte development through an unknown mechanism that needs further in-depth research (10). In addition, the essential roles of sclerostin on B cell maturation were further confirmed indirectly by the study about von Hippel-Lindau (Vhl), which modulates sclerostin expression *via* hypoxia response signaling pathway, implying the link between sclerostin and B cell development (11).

Taking advantages of aptamers-based high affinity and strong inhibitory roles to the target proteins, aptamers that could rival antibodies but are superior, have been used as essential approaches for diagnostic and therapeutic strategies in immune diseases (12). In some cases, they act as inhibitors by selectively and efficiently binding to targets; in other cases, thanks to their excellent targeting and subsequent endocytosis-mediated internalization capacities, aptamers could also be used as ideal carriers to deliver therapeutic agents for targeted therapy. In the context of multiple myeloma (MM) activities, a modified RNA aptamer, apt69.T, was synthesized to target B cell maturation antigen (BCMA), a critical factor in promoting plasma cells (PCs) survival, to inhibit MM activities (13). In addition, B cell antigens including CD19 and CD20 that are overexpressed on various B cell malignancies, are also suitable markers for aptamer targeting (14–16). Further, due to the cell-binding and internalization properties, a framework combining aptamers and therapeutic agents could be used for the therapeutic strategy for immune diseases (17, 18). Aptamers could also act as biotherapeutic agents by regulating cell cycles to achieve synergistic effects with drugs; the potential molecular basis of the process needs more experiments to elucidate (19). Interestingly, aptamers could also be used as an excellent tool for quality control of biosimilars due to their ability of detecting subtle conformational variations of molecules (20, 21). For diagnostic purposes, aptamer-imaging molecules conjugation complex would be formulated for convenient *in vivo* visualization (22, 23). Therefore, it is clear that aptamers can facilitate the development of novel therapeutic and diagnostic strategies for immune diseases.

## Sclerostin: An Inhibitor of Wnt Signaling Pathway

Sclerostin is a glycoprotein containing 213 residues with approximately calculated molecular weight 40 kDa. It is a well-known negative regulatory factor for bone-forming osteoblast, secreted by several cell types, primarily mature osteocytes (24). In the past debate, due to the unique cysteine-knot motif, sclerostin was classified as a member of neuroblastoma (DAN) protein family, which has been shown to have the ability to antagonize bone morphogenetic proteins (BMP) (25). Therefore, it was presumed that sclerostin inhibits bone growth through serving as a BMPs antagonist (26, 27), just like other members of the DAN family. Still, more recent research indicated that the sequence similarity between sclerostin and other members of the DAN family is somewhat limited (28). In addition, although sclerostin could bind to BMPs *in vitro*, the binding affinities were weak (26, 29). In order to address the unclear mechanism by which sclerostin antagonizes BMPs, van Bezooijen et al. discovered that sclerostin exerted its function through blocking Wnt signaling pathway but not acting as a BMPs antagonist (30). Mechanically, sclerostin was proven to inhibit Wnt signaling pathway through binding competitively to Wnt co-receptors low-density lipoprotein receptor-related proteins 5 and 6 (LRP5/6) (4, 5).

**Figure 1:**
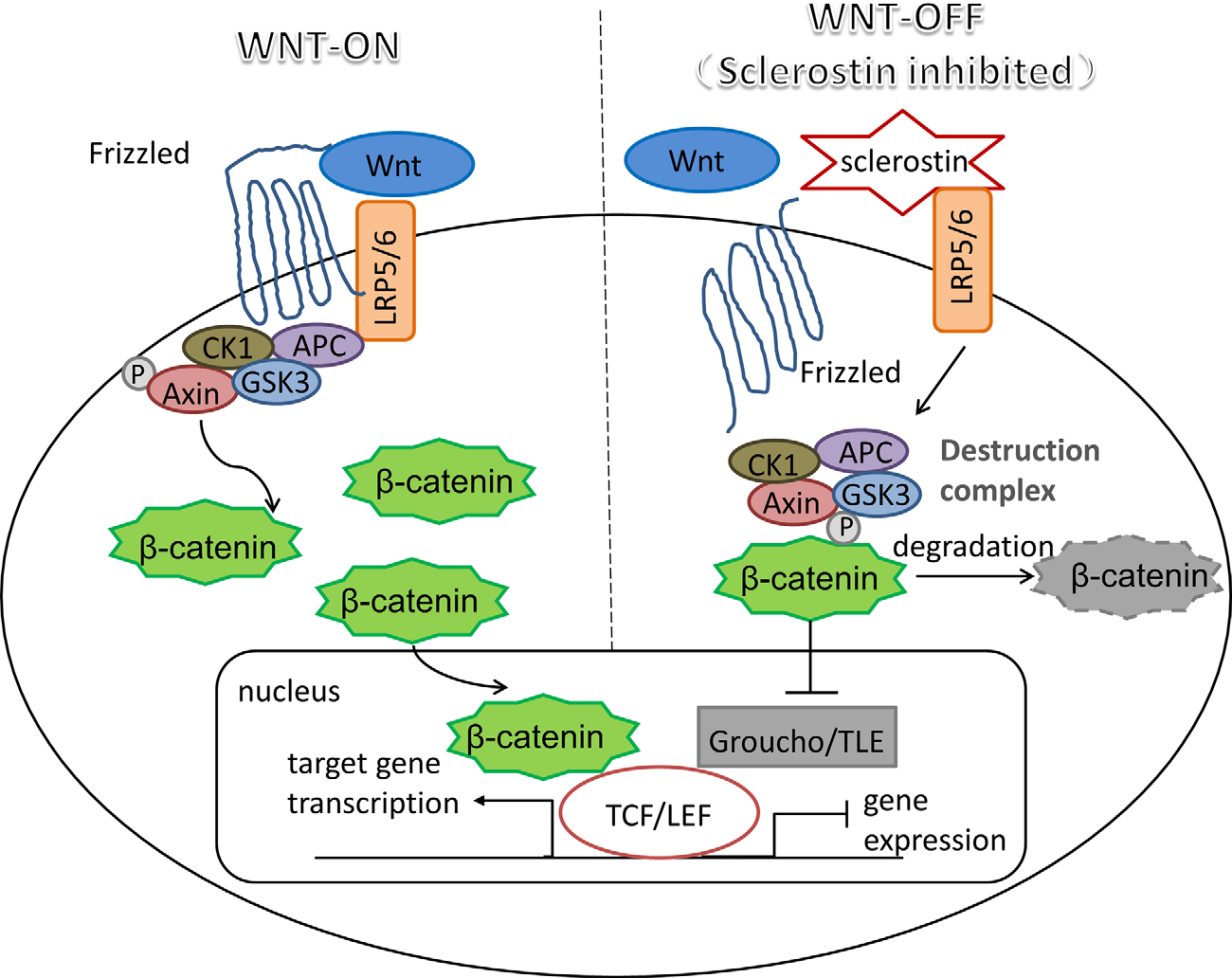
Sclerostin: an inhibitor of canonical Wnt signaling pathway. Wnt-LRP5/6-Fz complex elicits a molecular cascade that transfer Axin-mediated destruction complex, which is essential to *β*-catenin degradation, to the plasma membrane. Increased *β*-catenin moves into the nucleus, where it represents the molecular mechanism that *β*-catenin binds to TCF/LEF, which are the main partners of *β*-catenin to serve the transcriptional function of canonical Wnt signaling pathway. Sclerostin inhibits Wnt signaling by binding competitively to LRP5/6, thereby promoting the degradation of *β*-catenin mediated by destruction complex, resulting in the interaction between TCF/LEF and repressor Groucho/TLE proteins to halt the expression of target genes.

The mechanism of sclerostin as a Wnt inhibitor/antagonist blocking the Wnt signaling cascade has been demonstrated in a number of studies (31–34). As a critical pathway in almost every aspect of the developmental process and self-renewal in a number of adult tissues, Wnt signaling plays pivotal roles in changing the expression patterns of specific target genes (35, 36). An essential and heavily studied pathway in the Wnt signaling is the *β*-catenin dependent Wnt signaling, also known as canonical Wnt signaling, which modulates the stabilization and transfer of transcriptional co-activator *β*-catenin to nucleus. In the nucleus, *β*-catenin forms a complex with DNA-bound T cell factor proteins/lymphoid enhancer factor (TCFs/LEF), which are the leading partners of *β*-catenin to participate in developmental gene expression programs (37). The activation of canonical Wnt signaling pathway occurs as Wnt ligands bind with its co-receptors LRP5/6, which then form a complex with seven-pass transmembrane Frizzled (Fz or Fzd) proteins. The Wnt-Fz-LRP5/6 complex could then elicit a cascade of molecular events that inhibit the phosphorylation of *β*-catenin through Axin-mediated destruction complex, which consists of scaffolding protein Axin, adenomatous polyposis coli (APC), casein kinase 1 (CK1), and glycogen synthase kinase 3 (GSK3). As soon as Wnt-Fz-LRP5/6 complex is formed, the Axin-mediated destruction complex would translocate to the plasma membrane *via* being phosphorylated by other proteins within the destruction complex, thereby inhibiting the phosphorylation of *β*-catenin (38, 39). Therefore, in the situation of the Wnt signaling blocking, cytoplasmic *β*-catenin is degraded continuously under the influence of the destruction complex, since the phosphorylation of *β*-catenin creates a binding site that could be recognized by E3 ubiquitin ligase, which is responsible for the subsequent proteasomal degradation of *β*-catenin (40). Interestingly, TCF/LEF would combine with the repressor Groucho/TLE proteins when *β*-catenin is missing, which promotes histone deacetylation and chromatin compaction, thereby acting as a transcriptional repressor for the expression of target genes (41, 42). Therefore, when sclerostin competitively binds to LRP5/6 on the plasma membrane, it would result in significantly reduced *β*-catenin stability, thereby inhibiting the expression profiles of Wnt target genes (shown in **Figure 1**).

Numerous studies have supported the function of sclerostin in the canonical Wnt signaling pathway; several studies also showed that it could execute action in non-canonical Wnt signaling. For instance, sclerostin inhibits both the canonical Wnt signaling and the c-Jun N-terminal kinase (JNK) pathway, which is categorized into non-canonical Wnt signaling pathway, in osteoarthritis (43). In addition, similar with sclerostin, Dickkopf-1 (Dkk1), another inhibitor of the canonical Wnt signaling pathway, has also been proven to participate in the canonical Wnt signaling pathway, and to promote the *β*-catenin independent Wnt signaling in many types of cancers (44–46). But so far, the research about the roles of sclerostin in the non-canonical Wnt pathway in cancers are still lacking, which deserves more attention and effort of further exploration.

## The Roles of Sclerostin in Modulating the Immune Cells

### Effects of Canonical Wnt Signaling on B Lymphocyte

B lymphocytes are capable of generating immunoglobulins (Igs) to develop an antibody response to specific antigens when combating an infection (47). Together with T lymphocytes, these cells comprise the adaptive immune system. In a tightly ordered process, B cells first originated from hematopoietic stem cells (HSCs), then mature in fetal liver and bone marrow and finally reach secondary lymphoid organs. B lymphopoiesis in bone marrow relies heavily on assembly of the functional B-cell antigen receptors on the surface through different combinations of gene segments, known as V(D)J recombination, which is a crucial part of proper lymphocyte development (48). The developmental process includes several stages beginning with pro-B cells, to pre-B cells, immature B cells, and finally to mature B cells (49).

Studies on mice and human suggest that canonical Wnt signaling cascade is involved in the B cell development although the exact roles of Wnt signaling are not consistent in different microenvironment, which are summarized in **Table 1**. The expression of lymphocyte enhancer factor -1 (LEF-1) in developing B cells, which is a member of the TCF/LEF-1 transcription factors in Wnt signaling cascade, indicates the possibility that Wnt signaling might participate in the proliferation and/or differentiation of lymphoid cells. To address this question, Reya et al. examined proliferation, survival, and differentiation of B cells in LEF-1-deficient mice. They found that the absence of LEF-1 lead to defective pro-B cell proliferation and survival but not differentiation. Further, the potential molecular basis of this finding might be the increased apoptosis of B cells due to up-regulated *fas* and *c-myc* transcription, which could trigger cell death. Moreover, incubation of fetal liver pro-B cells with Wnt3A conditioned medium, which could activate canonical Wnt signaling cascade, lead to enhanced pro-B cell proliferation. Therefore, the results suggest a novel role of LEF-1-dependent Wnt signaling pathway in normal B cell proliferation (6). In addition, FZD9−/− mice, which also implicated the blockade of Wnt signaling, elicited pronounced defects in developing B cells in bone marrow, particularly pre-B cells. The above reports established that Wnt signaling cascade plays a central role in B cell development (7). However, in contrast to the findings made in murine pro-B cells from fetal liver, in the case of human bone marrow, it is found that B lymphopoiesis was inhibited by Wnt3A stimulation. In addition, this inhibitory effect was blocked by the Wnt antagonists sFRP1 or Dkk1. These results suggest that the canonical Wnt pathway acts as a negative regulator of proliferation potential of B cells in human bone marrow (8). The divergent results about positive and negative influence of Wnt signaling on murine and human B cells might be explained by the distinction in species and/or microenvironment between fetal liver and adult bone marrow, which needs further exploration.

**Table 1:** Effects of Wnt signaling pathway on B cells.

Effects on B cells	Ref
LEF-1-deficient mice lead to defective pro-B cell proliferation and survival but not differentiation	(6)
FZD9−/− mice elicit defects in developing B cells in bone marrow, particularly pre-B cell	(7)
B lymphopoiesis is inhibited by activation of canonical Wnt signaling pathway in human bone marrow	(8)

### Effects of Sclerostin on B Lymphocyte

In addition to the bone resorption and formation mediated by a variety of cytokines produced by T and B lymphocytes, osteoblast lineage cells also support hematopoietic cell survival and differentiated descendants such as B cells. Recently, the cross-talking between hematopoietic cells and bone cells has been an active area of investigation (50–52). Cain et al. were the first to explore the influence of sclerostin on the immune system. They found that sclerostin loss-of-function mice showed not only significantly elevated activity of osteoblast, but also altered normal B lymphocyte development through promoting B cell apoptosis. Absence of sclerostin expression in hematopoietic cells and any B cell population implied that the substantial B defects in sclerostin^−/−^ mice results from a non-cell autonomous effect, a result which was confirmed by reciprocal sclerostin^−/−^➞WT and WT➞ sclerostin^−/−^ chimeras studies. The transplantation of WT bone marrow into sclerostin^−/−^ recipients was followed by a decrease in B cells, whereas reciprocal sclerostin^−/−^➞WT was not. The results supported the idea that B-cell defects are not results from the changes in B-cell themselves, but from the alterations in the bone microenvironment. Low levels of CXCL12, an essential B cell growth-stimulating factor, were also observed in sclerostin^-/-^mice, compatible with B defects. The results match with another study that illustrated the negative correlation between the activation of Wnt signaling and CXCL12 levels (53). Therefore, the results suggest that sclerostin belongs to a group of factors that play critical roles in both bone formation and immune system. Interestingly, that known Wnt target genes *Lef-1* and *Ccnd1* expression pattern remains unchanged implies sclerostin plays a novel part to support B cell development in bone marrow independent of direct influence of Wnt signaling pathway on B cells. It might be explained by the indirect role of sclerostin on the mesenchymal stem cells (MSCs), a kind of stromal cells, which have been found to express CXCL12. Therefore, whether a causative link exists between sclerostin, MSCs, CXCL12, and B development remains unknown that needs further investigation (9, 54, 55). Moreover, depletion of B cells was only occurs in bone marrow but not the spleen. Under normal conditions, a majority of B cells in bone marrow are plasma cells that play a critical role in fighting an infection through rapid release of antigen-specific antibodies. Recirculating B cells migrate back to the bone marrow after stimulation in secondary lymphoid organs. The clear reduction of recirculating B cells in bone marrow suggests that bone marrow environment is not conductive for maintenance of B cells even after the completion of activation of the B cells in the periphery. Therefore, immunodeficiency might occur in the patients receiving sclerostin antibodies treatment, which should deserve more attention.

However, whether sclerostin in different osteolineage cells contributes differently to the B lymphocytes development is still a significant knowledge gap. Subsequently, Yee et al. reported that conditional loss of sclerostin in different osteolineage cells induced differentially altered B lymphocyte development. The results showed that sclerostin in mesenchymal stem cells (MSCs) and osteoblasts is essential for B cell development, while sclerostin in mature osteocytes does not play a critical role in B cell survival. The findings are consistent with the previous hypothesis that sclerostin might mediate B cell development which depends on MSCs and CXCL12 that needs to be tested through further studies. Sclerostin-deficiency in MSCs (*Prx1-Cre)* mice displayed abnormal accumulation of cells lacking IgM and IgD. The presence of IgM and IgD is the feature of mature B cells, suggesting sclerostin in *Prx1+* cells plays a role at the later stages of B cell progression. On the other hand, sclerostin-deficiency in mature osteoblasts (*Coll-Cre)* delayed the early phase of B cell maturation due to significantly high proportion of IgM^−^IgD^−^ cells in B progenitors at B220^+^CD43^high^ stage. However, whether CXCL12 expression is also changed in MSCs and osteoblasts and the underlying mechanism still remains poorly understood, which needs further experimental studies to elucidate (10).

Additionally, the critical role of sclerostin in B cell development was further examined indirectly by the study in which von Hippel-Lindau (Vhl) depletion contributes towards concomitant high bone mass and impaired B cell development through promoting Wnt signaling pathway and reducing the expression of sclerostin (11). According to the variation of oxygen levels, proline hydroxylated hypoxia-inducible factor (HIF) acts as a transcription factor *via* the interaction between HIF-*β* and one of HIF-*α* isoforms (HIF-1*α*, HIF-2*α*, and HIF-3*α*). Under normoxic conditions, HIF1*α* would be targeted by E3 ligase complex Vhl and ultimately degraded *via* the proteasome. In contrast, under hypoxic state, increased HIF1α accumulation is achieved by inhibiting prolyl hydroxylation. The HIF complex would then transfer into the nucleus acting as a transcription factor. Therefore, in Vhl-knockout mice, HIF1*α* is stabilized. In addition, reduction in sclerostin and concomitant increase in activated *β*-catenin was observed by immunocytochemistry, which indirectly further examines the link between sclerostin and B cell development (56). For example, absolute numbers of CD45^+^ hematopoietic cells and CD19^+^ B lymphocytes were significantly reduced. Additionally, B cell maturation was also disrupted in the spleen through reducing mature B cells (CD19^high^B220^high^) with abnormally immature phenotype (IgM^+^IgD^low^).

Although sclerostin has been proven to participate in normal B cell development and differentiation, in the case of immune diseases, the research on the applications of monoclonal sclerostin antibody is still mainly limited to its influence on bone formation. For example, elevated levels of sclerostin in serum and osteocytes were reported in multiple myeloma (MM), an aggressive lethal hematologic disease accompanied with detectable severe bone destruction (57, 58). As expected, combining anti-sclerostin antibody with chemotherapy could control MM growth and reverse osteolysis (59). Surprisingly, several *in vivo and vitro* studies reported that sclerostin inhibition alone did not affect tumor burden, an issue that needs further research through more experimental studies (60).

Taken together, the above research studies imply that people receiving any therapies directed at anti-sclerostin might suffer from B cell defects. Still, no data from clinical trials of the romosozumab, a monoclonal antibody that targets sclerostin, is available about its effects on the immune functions of patients (61, 62). Moreover, knowledge about the roles of sclerostin in immune diseases is still limited, which needs further in-depth research.

### Effects of Sclerostin on T Lymphocyte

Although Cain et al. illustrated that the absence of sclerostin results in B cell-specific defects but not the difference of cell numbers in T lymphocytes, natural killer cells, monocytes, granulocytes, and erythroid cells, You et al. demonstrated that sclerostin is necessary for inducing T helper 17 (Th17) cell differentiation, which is responsible for bone resorption, through promoting the levels of IL-6 and TFG-*β* that are related to Th17 differentiation. In addition, sclerostin inhibits the differentiation of regulatory T (Treg) cells *via* reducing the expression of IL-10 and Foxp3, which play an essential role in Treg cell development (63). Given that Th17 and Treg cells plays vital roles in inflammatory bone diseases, the research provides valuable hints about the therapeutic strategy for this kind of disease involving the imbalance of Th17 and Treg cells development. Taken together, the exact roles of sclerostin in immune cells are summarized in **Table 2**.

**Table 2:** Effects of sclerostin on immune cells.

Effects on immune cells	Affected immune cell	Ref
absence of sclerostin inhibits normal B lymphocyte development through promoting B cell apoptosis		(9)
conditional loss of sclerostin in different osteolineage cells indicated differentially altered B lymphocyte development	B cell	(10)
von Hippel-Lindau (Vhl) depletion showed the enhanced Wnt signaling activity through inhibiting sclerostin expression with subsequent impaired B cell development		(11)
sclerostin is necessary in inducing T helper 17 (Th17) cell differentiation, and inhibiting the differentiation of regulatory T (Treg) cells	T cell	(63)

## Aptamers-Based Research on Immune Diseases

Aptamers are single-stranded (ss) oligonucleotide sequences (DNA/RNA) with a length of approximate 25–80 bases that are capable of binding to a variety of specific target molecules by virtue of their unique three-dimensional structures. Considering their high binding affinity and specificity, ease in production, modification flexibility, minimal batch-to-batch variability, low immunogenicity, *etc*., in recent years, aptamers have gained extensive research attention as a potent alternative of antibodies (12). An aptamer’s selection technology, known as Systematic Evolution of Ligands by Exponential Enrichment (SELEX), was first developed in 1990 by two laboratories (64, 65). The fundamental selection cycle requires three critical steps: 1) incubating a target with a chemically synthesized oligonucleotide library containing randomized sequences (DNA/RNA); 2) removing unbound sequences and splitting bound sequences from target; 3) amplifying the bound sequences by PCR. In the case of RNA aptamer selection, additional reverse transcription into DNA is necessary (66). A number of selection cycles are then performed until the sequence with desired affinity is obtained. Several critical modifications are introduced to achieve long-lasting action time through overcoming nuclease degradation (67). On the one hand, aptamers act as inhibitors that can interfere with the normal function of a target protein, mimicking the functional properties of monoclonal antibodies. On the other hand, some aptamers will internalize after binding to receptors on the cell membrane, so that they can act as vehicles to deliver drugs, imaging agents, microRNAs, small interfering RNAs (siRNA), *etc*. Therefore, since aptamers have both inhibitory and carrier capabilities, significant clinical applications have been developed to realize their diagnostic and therapeutic potentials (68–71).

Multiple myeloma (MM) is characterized by abnormal accumulation of malignant plasma cells (PCs) that produce immunoglobulins (72). B cell maturation antigen (BCMA) is exclusively expressed on the surface of terminally differentiated B cells and is highly expressed on malignant PCs (73). Mechanically, BCMA promotes long-survival of PCs through binding to their specific ligands, B cell activating factor (BAFF) and the proliferation-inducing ligand (APRIL), which could trigger the activation of downstream nuclear factor kB (NF-kB) pathway (74, 75). As a result, monoclonal antibodies (mAbs) targeting BCMA have been used as effective therapeutic tools for MM (76, 77). Considering the various advantages of aptamers over antibodies, Catuogno et al. selected a 2′Fluoro-Pyrimidine modified RNA aptamer, apt69.T, that could effectively bind to BCMA-enriched myeloma cells with excellent serum stability. Further, subsequent internalization of apt69.T into the cells makes it a suitable tool for direct targeting and delivery of therapeutics (13). Overexpression of BAFF receptor was also observed on the surface of numerous B cell malignancies (78). Therefore, Zhou et al. formulated a BAFF aptamer–siRNA conjugation complex through a “sticky bridge” for B-cell lymphoma therapy (17). In conclusion, aptamer-based targeted therapies and drug delivery system provide a framework for the future therapeutic strategy for immune diseases.

The B-lymphocyte antigen (CD20) is another suitable candidate for recognition of B cells, since it expresses on the surface of the almost all the precursor and mature B lymphocytes, even malignant B cells, except in normal plasma cells (79). CD20 acts as a voltage-independent Ca^2+^ channel that regulates the activation and proliferation of B-cells through mediating the concentration of Ca^2+^ and triggering tyrosine kinase signaling pathways (80, 81). The significant outcomes of rituximab, an anti-CD20 monoclonal antibody, have been achieved in various B cell malignancies, such as non-Hodgkin’s lymphoma, Burkitt’s mature B-cell lymphoma, and chronic lymphocytic leukemia (CLL) (82, 83). However, limitations of antibodies including their thermal instability and immunogenicity underscore the urgent need to develop appropriate aptamers that could be used as an alternative and effective tool for therapeutic practices. After 10 rounds of SELEX screening, a panel of candidate aptamers was generated. Further analysis of characterization demonstrated that the most thermodynamically stable aptamer AP-1 has the strongest binding affinities with CD20 (14). However, in order to accelerate the clinical translation of therapeutic aptamers, inherent physicochemical characteristics and safety should be evaluated through further in-depth investigation.

Interestingly, aptamers can also act as an efficient quality control tool for biosimilars (products with high similarity of reference biological medicines) due to their ability of detecting subtle conformational variations of biologics. Although the amino acid sequences of biosimilars are identical to that of the originators, the biosimilars might still be different from the reference products due to post-translational modifications, which could be induced by highly complex production process. Therefore, it is necessary to evaluate the detailed characterization, such as 3D shape, which is crucial to detecting the differences between biosimilars and originators. However, there are only a limited number of laborious methods, such as NMR, X-ray crystallography, or monoclonal antibodies that can specifically target biologics. In addition, difficulties and high cost of producing appropriate antibody panels greatly hinder the development and approval of biosimilars. In order to improve the quality assessment for properties of biosimilars, aptamers were generated to monitor conformational similarities of biosimilars and reference products. Wildner et al. screened a first panel of anti-CD20 antibody rituximab-specific aptamers that could detect conformational variations. In addition, the selected aptamers also demonstrated the changes in structure upon thermo or UV exposure of rituximab. In the study, the authors chose a 40 nucleotide random part instead of general oligonucleotide libraries with a length of 20–80 nucleotides to obtain stable structures with high affinity to the target protein (20). In addition to recognition of native state of rituximab, the same team further generated six high-affinity DNA aptamers capable of selectively recognizing the distinct structural determinants of heat-treated rituximab prior to precipitation. None of the reaction was observed with the antibody in its native state or when being exposed to other physical stresses (21). Hence, aptamers could be used as a suitable sensor for detecting structural variations of biologics, with the potential for stringent biopharmaceutical quality control. They can also serve as a useful tool for studying the unfolding process of proteins when stresses exposure occurs.

Following the success of CD20-targeted antibody rituximab, other attempts are also made to develop novel therapies for B cell malignancies. High and stable levels of CD19 were also detected on the surfaces of various B cell malignancies, such as acute lymphocytic leukemia, chronic lymphocytic leukemia, and Non-Hodgins lymphoma (84). Mechanically, CD19 enhances the chance of B cell survival by triggering B cell antigen receptor (BCR) signaling (85). Further, CD19-targeted chimeric antigen receptor (CAR)-modified T cell therapy achieved unprecedented success in clinical trials (86). Such evidence suggests that CD19 is of great significance as a diagnostic marker and therapeutic target for B cell malignancies. Hu et al. selected a first CD19 aptamer (LC1) to specifically target CD19-positive lymphoma cells but not CD19- negative cell lines. Furthermore, an aptamer–doxorubicin complex (Apt–Dox) was formulated and used to selectively deliver doxorubicin, a cytotoxic drug, to CD19-positive lymphoma cells *in vitro*. Free doxorubicin could diffuse into both the CD19-positive and negative cells. However, through targeted delivery of drug into CD19 positive cells *via* Apt–Dox, the problem of drug toxicity to negative cells can be solved, which has the potential for the development of targeted therapy. Additionally, although LC1 aptamer could be used as an important ligand with diagnostic or therapeutic potential through conjugating imaging contrasts or various drugs; however, the detailed mechanism of how Apt–Dox enter cells was not well known. In order to facilitate the development of other aptamer–drug conjugates, more in-depth research about such mechanism of internalization is needed (15). Since drug resistance always occurs with chemotherapy treatment, the elevated levels of P-glycoprotein (P-gp) on the cell surface would result in drug resistance through limiting drug entry into cells (87). Another protein, B-cell lymphoma 2 (Bcl2), acting as an anti-apoptotic factor on the mitochondrial membrane, also contributes to the drug resistance through inhibiting cell death (88). Therefore, Pan et al. constructed a multifunctional DNA origami-based carrier, a promising candidate for tumor imaging, with both doxorubicin and two different antisense oligonucleotides (ASOs) that target P-gp and Bcl2, for enhancing efficacy of treatment (16). This strategy reveals the potential of combining chemotherapy and oligonucleotides in aptamer-based targeted therapy. In order to study the structural–affinity relationships between aptamers and the target proteins, Danquah et al. discovered an ‘aptamer walking’ mechanism through molecular dynamics (MD) simulation. They constructed a CD19–aptamer complex based on data available in the protein data bank. The results indicated that aptamer molecules could gradually adjust its configuration and shift to a favorable binding position, whilst CD19 remains relatively stable. The aptamers and their stable binding-poses relative to CD19 might be used as suitable templates in designing potential aptamer molecules. In addition, the structural approach adopted in this study provides a novel direction for searching various aptamer molecules for specific targets in future (89).

The cytotoxic effects of doxorubicin on lymphoma depend on Topoisomerase II alpha (TopIIA), a DNA repair enzyme complex. The complex plays a key role in repairing DNA damage due to its ability to relax supercoiled DNA (90). Although anthracyclines, including doxorubicin and etoposide, have shown great clinical significance in the treatment of large B-cell lymphoma (DLBCL), the response of the cancer cells to the therapy vary considerably across cases (91). Previous studies reported that elevated expression of nucleolin in B-cell lymphoma cell lines, involving DLBCL, compared to normal B cells (92). Although nucleolin was proven to be associated with several key DNA repair proteins, the functions of nucleolin in DNA damage response are unclear (93). Jain et al. found that nucleolin plays a novel modulatory role in DNA repair when binding to TopIIA. The results demonstrated that the nucleolin–TopIIA interaction prevents the killing effects of TopIIA targeting agents on DLBCL cells by facilitating DNA damage repair instead of cleavage. In other words, silencing of nucleolin could enhance the TopIIA targeting agent-induced DNA damage and apoptosis of DLBCL. Consequently, combining nucleolin inhibitor (aptamer AS1411) with doxorubicin greatly reduces the survival chance of DLBCL cells by compromising DNA repair capabilities provided by TopIIA. Thus, in order to improve the efficacy of TopIIA targeting agents, combination of agents and nucleolin-targeted aptamers might be a promising solution (18).

Protein tyrosine kinase 7 (PTK7) membrane receptor was reported to participate and up-regulate in the progression of various cancers, including hematological malignancies (94). Also, PTK7 expression promotes cultured leukemia cells resistance to anthracycline-induced apoptosis (95). Sgc8-c aptamer, the truncated form of original aptamer Sgc8, was used to target PTK7 as a therapeutic tool with similar binding affinities of Sgc8 (96, 97). To serve as the radiolabeled probe for theranostic purpose, Sgc8-c aptamer was labeled with ^67^Ga and metal chelator NOTA conjugation with good biodistribution and molecular imaging both *in vivo* and *vitro* evaluation (23). In addition, other PTK7-targeting aptamer-fluorescent and -radiolabeled probes with fluorescent dye AlexaFluor647 and 6-hydrazinonicotinamide (HYNIC) chelator were also formulated for *in vivo* visualization (22). However, in order to further enhance tumor retention, additional chemical modifications have to be performed.

Interestingly, in addition to the cell-binding property, aptamers might also act as biotherapeutic agents by regulating cell cycles. Li et al. synthesized an ssDNA aptamer specifically targeting Maver-1 lymphoma cells with concomitant endocytosis-mediated internalization that triggered S-phase arrest. The induced arrest primed target cells for cytarabine chemotherapy, which primarily kills lymphoma cells at S-phase (19). Therefore, the synergistic killing effects achieved by the combination of aptamers and chemotherapeutic agents open a new avenue for precision therapy. However, although some efforts about the detection of expression of several key proteins in cellular signaling pathways have been made, the exact mechanism through which internalized aptamers regulate the intracellular signaling pathways remains unclear, which needs further investigation.

To sum up, the review in this section suggests that we could use aptamers as promising research tools to develop therapeutic and diagnostic strategies for immune diseases based on the inhibitory or carrier properties of the aptamers (shown in **Table 3**).

**Table 3:** Aptamers-based research on immune diseases.

Functions of aptamers	Targets/modifications/therapeutic agents	Ref
**Inhibitors**	B cell maturation antigen (BCMA) 2′Fluoro-Pyrimidine modification	(13)
	CD20	(14)
**Delivery carriers**	B cell activating factor (BAFF) + siRNA	(17)
	CD19 +doxorubicin +doxorubicin+ASOs targeting P-gp and Bcl2	(15, 16)
	Nucleolin + doxorubicin	(18)
**Diagnostic agents**	PTK7 +67Ga+metal chelator NOTA +AlexaFluor647 +6-hydrazinonicotinamide (HYNIC)	(22, 23)
**Others**	anti-CD20 antibody rituximab Maver-1 lymphoma cells	(19–21)

## Conclusions and Future Perspectives

As a negative regulator of bone growth, sclerostin, a Wnt signaling antagonist based primarily on binding competitively to Wnt co-receptors LRP5/6, received not only extensive attention for its therapeutic effects in bone diseases, but also plays critical roles on development and differentiation of immune cells, especially B cells.

On the one hand, multiple studies have reported that sclerostin is indispensable for B cell survival and development. Sclerostin loss-of-function mice showed B cell defects through enhanced B cell apoptosis, concomitant with reduced levels of CXCL12, a critical B cell growth-stimulating mediator. Interestingly, unchanged Wnt target genes *Lef-1* and *Ccnd1* expression pattern implies a novel role of sclerostin in supporting B cell functions independent of Wnt signaling pathway, which needs further investigation. In addition, conditional loss of sclerostin in different osteolineage cells demonstrated differentially altered B lymphocyte development. The essential influence of sclerostin on B cell maturation was further confirmed indirectly by the study about von Hippel-Lindau (Vhl), which modulates sclerostin expression *via* hypoxia response signaling pathway, revealing the relationship between sclerostin and B cell development. In spite of the effects of sclerostin antibodies in immune diseases that mainly focus on bone formation, the roles of sclerostin in these immune diseases need further investigation to facilitate the development of sclerostin-based therapies for immune diseases.

Currently, a variety of aptamers have been generated and used in various studies of immune diseases. Thanks to their excellent targeting and subsequent endocytosis-mediated internalization capabilities, aptamers act as inhibitors or valuable carriers for targeted therapy. In order to accelerate the clinical translations of these therapeutic aptamers, aptamers should be made to improve factors including physicochemical characteristics, modifications, safety, *etc*.

In summary, although the underlying mechanism of sclerostin in immune diseases has not yet been fully elucidated, its roles in normal immune cell development, especially B cells, have become increasingly apparent. It is clear that aptamers are competent tools for subsequent progress in translational pharmacology based on the sclerostin functional study. The utilization of aptamers might facilitate the development of novel anti-immune diseases therapeutic and diagnostic strategies based on sclerostin.

## Author Contributions

MS wrote the manuscript. FL, XW, and YY helped in revising the manuscript. MS, ZC, and LW contributed in figure designing. FL, AL, and GZ supervised the preparation of the manuscript. All authors contributed to the article and approved the submitted version.

## Funding

This study was supported by the National Key Research and Development Program of China (2018YFA0800804), the Interdisciplinary Research Matching Scheme Hong Kong Baptist University (RC-IRMS/15-16/01), the Hong Kong General Research Fund (12101018, 12102518, 12102120), Theme-based Research Scheme Hong Kong Research Grants Council (TRS/RGC T12-201-20-R), and the National Natural Science Foundation Council of China (81703049).

## Conflict of Interest

The authors declare that the research was conducted in the absence of any commercial or financial relationships that could be construed as a potential conflict of interest.
